# Australian Women in the Perinatal Period During COVID-19: The Influence of Self-Compassion and Emotional Regulation on Anxiety, Depression, and Social Anxiety

**DOI:** 10.3390/healthcare13020120

**Published:** 2025-01-09

**Authors:** Kayla Cutajar, Glen William Bates

**Affiliations:** Department of Psychological Sciences, Swinburne University of Technology, Hawthorn, VIC 3122, Australia; kaylacutajar96@gmail.com

**Keywords:** perinatal women, depression, anxiety, social anxiety, self-compassion, emotional regulation

## Abstract

**Objectives**: This study examined how self-compassion and emotional regulation strategies have influenced perinatal anxiety, depression, and social anxiety during COVID-19. **Methods**: A probabilistic sample, determined by convenience criteria of 265 Australian perinatal women completed an online survey containing measures of depression, anxiety, social anxiety, COVID-19 experiences, self-compassion, and emotional regulation strategies. **Results**: As hypothesised, correlation analyses showed that self-compassion and adaptive emotional regulation strategies were negatively related to anxiety, depression and social anxiety, and maladaptive strategies were positively related. Contrary to predictions, COVID-19-related experiences showed little relationship with mental health outcomes. Parallel mediation analyses showed that self-compassion negatively predicted depression and anxiety and was partially mediated by specific emotional regulation strategies. For social anxiety, self-compassion was fully mediated by emotional regulation strategies. Different emotional regulation strategies were significant mediators of the relationship between self-compassion and each mental health outcome. **Conclusions**: The findings suggest that reinforcing self-compassion and addressing certain emotional regulation deficits is important in alleviating mental health symptoms among perinatal women.

## 1. Introduction

Women in the perinatal period are known to have a higher risk of experiencing mental health concerns [1] and are vulnerable to environmental stressors [2]. Research shows that one in five perinatal women experiences anxiety and one in ten experiences depression [3,4]. Moreover, perinatal women were found to be more vulnerable to psychological distress during the COVID-19 pandemic with research suggesting that the COVID-19 pandemic has exacerbated the risk of mental health issues for perinatal women globally [5,6,7,8]. Perinatal women understandably feared infections for themselves and their infants during COVID-19 as emerging research suggests potential effects of maternal COVID-19 infection on intrauterine development, including an increased risk of fetal growth restriction, particularly with early pregnancy infections [9] and increased risk of gestational hypertension in pregnant women with prolonged infection [10]. Additionally, prenatal and postnatal women experienced heightened psychosocial stress due to pandemic-related restrictions, such as limited social support and hospital access and were under increased stress due to mandatory COVID-19 adjustments (i.e., working restrictions, hospital and appointment restrictions to mother only, reduced social supports [11]). As in other countries, perinatal women were identified as a vulnerable group in the community despite mixed evidence about their actual degree of risk, exacerbating their concerns about infection for themselves and their babies [12].

In Australia, national surveys conducted in 2020 showed a 51% increase in calls to perinatal mental health support lines [13] with 43% of callers reporting their mental health had been impacted by COVID-19 [14]. A meta-analysis [11] looked at studies across Asia, Europe and North America on the prevalence of perinatal anxiety and depression symptoms during the COVID-19 pandemic and found that perinatal anxiety almost tripled compared to levels reported in previous meta-analyses (e.g., [15]). Although these statistics raise concerns about the vulnerability of perinatal women and infants, most studies have targeted pregnant women and have been conducted outside Australia. Additionally, while anxiety and depression have received considerable attention, social anxiety emerged as a major problem in the community during the COVID-19 pandemic [16,17]. Therefore, this study sought to better understand how depression, general anxiety and social anxiety presented in Australian perinatal women as well as to explore the relationship with their COVID-19 experience.

### 1.1. Social Anxiety and Perinatal Women

A systematic review by Kindred and Bates [17] found that the incidence of social anxiety among women had increased during the COVID-19 pandemic and even prior to the onset of COVID-19, social anxiety had been recognised as a “hidden epidemic” [18]. Interestingly, women showed over double the rates of social anxiety prevalence compared to men in the Australian Bureau of Statistics’ 2022 review [16]. Despite this, there is currently no specific research on social anxiety in perinatal women. A systematic review by McCarthy et al. [19] indicated, however, that perinatal women are likely to fear social scrutiny due to the pressure of meeting social norms to be a “good mother” and this is related to the presence of anxiety in social situations, that is, social anxiety. Moreover, COVID-19 restrictions and isolations have likely increased fear of meeting such expectations in perinatal women and provided them with fewer opportunities to build their social confidence as a mother. Taken together, this suggests there may be higher rates of social anxiety within the perinatal population that have gone unrecognised, and which are likely to have made these women more vulnerable during the COVID-19 pandemic. This underscores the need to better understand social anxiety in perinatal women and the impact of the COVID-19 pandemic.

### 1.2. Emotional Regulation for Perinatal Women

Emotional regulation has been defined as the processes whereby individuals seek to influence their experience of emotions and their expression of emotion [20]. Considering the increase in anxiety and depression, and possibly social anxiety, in the perinatal population during the COVID-19 pandemic, it seems important to understand which emotional regulation strategies could have influenced mental health outcomes during that time. Maladaptive behavioural emotional regulation (e.g., distraction and numbing behaviours like comfort eating) were shown to be closely associated with anxiety and depression symptoms among perinatal women [21]. Yet, there is currently no data on the relationships between specific inner emotional regulation strategies and perinatal mental health. However, research on the general population shows that broad emotional regulation strategies such as self-compassion are good predictors of mental health outcomes [22,23,24].

Neff’s framework of self-compassion as a broad form of emotional regulation is clearly associated with mental health outcomes [25] and has been investigated in perinatal research [26,27,28]. Neff [29] defined self-compassion as a person’s ability to be sensitive to their own experience of suffering. This inward concern for the self manifests in three general ways: self-kindness (vs. self-judgement), awareness of common humanity in painful experiences (vs. feeling isolated in suffering), and mindfulness (vs. overidentification with difficult experiences). Felder et al. [26] found that self-compassion scores explained 10–24% of the variation in perinatal anxiety and depression, with high self-judgment, isolation, low self-kindness, and common humanity associated with higher anxiety and depression. COVID-19-related studies also show that higher levels of self-compassion predict less impairment in mother–infant bonding, reduced perinatal depressive and anxiety symptoms and lower birth-related fear [27,30].

A study by Carona et al. [28] examined the role of self-compassion and mediating emotion regulation difficulties in perinatal depression and anxiety. They found that higher self-compassion predicted lower perinatal anxiety and depression directly and indirectly via general emotion regulation difficulties. These specific difficulties included non-acceptance of emotions, lack of emotion awareness and clarity, limited access to emotional regulation strategies and engaging in goal-directed behaviour and impulse control. A similar relationship between emotion regulation and self-compassion was shown in the prediction of social anxiety symptoms within a general student sample [22]. As these studies used data collected before the COVID-19 pandemic, they do not indicate how COVID-19 may have influenced these relationships. The present study built on these studies by including assessments for possible impacts of the COVID-19 experience.

Some research sheds light on the relationship between perinatal distress during COVID-19 and specific emotional regulation strategies such as information seeking, self-efficacy, comfort eating, and self-blame [3,21,31,32,33,34,35]. A study by Di Paolo [36] explored how resilience, tolerance of uncertainty and cognitive appraisal of the pandemic’s consequences affected postpartum anxiety and found negative cognitive appraisals predicted higher postpartum anxiety during COVID-19. Nevertheless, the specific emotional regulation strategies that most strongly influence social anxiety, anxiety and depression symptoms in perinatal women remain largely unknown. In general population studies, Chesney et al. [24] and Aldao et al. [37] established that adaptive emotional regulation strategies (i.e., acceptance, cognitive reappraisal and problem solving) relate to lower symptoms of anxiety and depression; whereas maladaptive strategies (i.e., avoidance, emotion suppression and rumination) increase symptoms. Building on Di Paolo’s findings on cognitive reappraisal [36], it is important to understand whether the relationships of specific adaptive and maladaptive emotional regulation strategies and mental health outcomes are replicated in perinatal women.

### 1.3. The Present Study

The overall aim of this study was to examine how self-compassion, emotional regulation strategies and COVID-related experiences influence anxiety, depression, and social anxiety symptoms in Australian perinatal women. It was predicted that:

1. Higher self-compassion and adaptive emotional regulation factors (acceptance, cognitive reappraisal, problem solving) would negatively correlate with anxiety, depression, and social anxiety symptoms.
2. High COVID-19-related stress, lower self-compassion, and maladaptive emotion regulation factors (lack of emotional understanding, expressive suppression, rumination) would positively correlate with anxiety, depression, and social anxiety symptoms.

Further, based on previous studies [22,26,28], the relationship between self-compassion and mental health symptoms was expected to be mediated by adaptive and maladaptive emotional regulation strategies during COVID-19. COVID-19-perceived experiences were also expected to mediate the relationship between self-compassion and mental health symptoms because higher COVID-19-related concerns would likely reduce the influence of self-compassion on mental health. It was hypothesised that:

3. Adaptive emotion regulation (acceptance, cognitive reappraisal, and problem solving) would positively mediate the influence of self-compassion on anxiety, depression, and social anxiety symptoms.
4. Maladaptive emotional regulation (rumination, expressive suppression, lack of emotional understanding) and more negative COVID experience would negatively mediate the influence of self-compassion on anxiety, depression, and social anxiety symptoms.

## 2. Materials and Methods

### 2.1. Participants and Procedure

This study adopted a heterogeneous cross-sectional design, targeting women in various stages of the perinatal period from pregnancy or up to two years post-birth [38]. The study received ethics clearance from the Swinburne University Human Research Ethics Committee (approval number 20226567-10039). Data were collected between August 2022 and June 2023. Inclusion criteria mandated participants to be 18 years or older and residents of Australia. Recruitment ensued through targeted emails to women enrolled in perinatal mental health services, while an additional avenue involved unpaid advertisements on social media platforms and online mothers’ group pages. Swinburne University students within the perinatal period were also invited through the Research Experience Program portal, earning course credit for participation. The utilisation of diverse recruitment channels was aimed at obtaining a more comprehensive and unbiased representation of the population of Australian perinatal women. Invitations, disseminated through emails and advertisements, included a survey link providing project details and an informed consent process. Participants retained the right to withdraw consent at any point by abstaining from submission.

To ascertain an optimal sample size for a moderate effect size, an a priori G-power analysis was performed, indicating a requirement of 160 participants. In the end, 265 participants completed the online survey. The total sample comprised women, aged 18–56, from diverse regions in Victoria and other states of Australia. Most of the participants were postnatal, married, or in partnerships, with a range of career paths and varying degrees of involvement in therapy. Table 1 presents a detailed breakdown of participant characteristics.

### 2.2. Measures

#### 2.2.1. COVID-19-Perceived Concerns

COVID-19 Perinatal Perception Questionnaire (COVID-19-PPQ; [39]). The COVID-19-PPQ was developed in response to the first global lockdown in 2020 by surveying perinatal women on perceived COVID-19-related stress in the perinatal period. The questionnaire consisted of pregnancy (nine items, e.g., ‘it bothered me that my scheduled ultrasounds could not continue’) and postpartum (10 items, e.g., ‘I was afraid that my partner would be infected with COVID-19’) subscales, measured with a 4-point response from 1 (Completely disagree) to 4 (Fully disagree). Higher scores indicated greater agreement with an unpleasant experience. The COVID-19-PPQ was found to have adequate psychometric properties for both scales including appropriate reliability and adequate-excellent model fit.

#### 2.2.2. Mental Health

The Hospital Anxiety and Depression Scale (HADS; Zigmond and Snaith [40]). The HADS is a 14-item self-report scale that assesses depression and anxiety symptoms in the previous week and is the most widely validated anxiety measure in the perinatal population [41] and the depression measures have substantial overlap with the Edinburgh Perinatal Depression Scale [42]. The HADS comprised seven anxiety items (e.g., ‘I feel restless as I have to be on the move’), and seven depression items (e.g., ‘I feel as if I am slowed down’), answered on a 4-point Likert scale (from 0 to 3 with varied anchors). The total subscale scores range from 0 to 21 with higher scores indicating greater symptoms of anxiety and depression. The theoretical median is 10.5. A cut-off score of 11 or higher is indicative of clinical levels of anxiety and depression symptoms [40].

The Social Interaction Phobia Scale (SIPS; [43]). The SIPS is a combination of elements from two social anxiety scales, the Social Phobia Scale (SPS; [44]) and the Social Interaction Anxiety Scale (SIAS; [44]), both on a five-point rating scale. The total SIPS score is calculated by summing the social interaction anxiety (SIA) subscale (consisting of five SIAS items) the fear of overt evaluation (FOE) subscale (consisting of six SPS items), and the fear of attracting attention (FAA) subscale (consisting of three SPS items). The SIAS items are rated from 0 ‘Not at all characteristic or true of me’ to 4 ‘Extremely characteristic or true of me’, and the SPS items are rated from 0 = ‘Not at all characteristic or true of me’ to 4 = ‘Extremely characteristic or true of me’. Total SIPS scores can range from 0 to 44 with higher scores indicating greater social anxiety. The theoretical median is 22 and the cut-off score for clinical levels of social anxiety is 21 [43]. The SIPS has enhanced utility and excellent internal consistency in clinical (a = 0.95) and good internal consistency for non-clinical populations (alpha = 0.87; [45]).

#### 2.2.3. Emotional Regulation

Due to the paucity of emotion regulation research on the perinatal period, measures of adaptive and maladaptive regulation strategies were selected based on previous research and a survey of perinatal clinicians. Eight perinatal mental health clinicians provided their clinical opinion regarding the prevalence of commonly observed emotional regulation strategies within the perinatal population. The clinicians were provided with a list of regulation strategies taken from various emotion regulation scales to rate what strategies they noticed more so [46,47,48,49]. Similarly to research by Chesney, Aldao and colleagues [24,37], acceptance, problem solving, expressive suppression, avoidance and rumination were identified by the clinicians as commonly observed in perinatal women accessing mental health support during the COVID-19 pandemic. Though cognitive reappraisal was not suggested by the clinicians as common, it was also included given its prominence as a measure of adaptive emotion regulation in the literature [24]. It is possible that the clinicians completing the survey based their responses on initial presentation to mental health services rather than effective strategies that may need to be developed such as cognitive reappraisal.

Self-Compassion Scale-Short Form (SCS-SF; [50]). The SCS-SF is a 12-item, shortened version of the 26-item Self-Compassion Scale [29]. The short form measures each of the six components of self-compassion identified by Neff [29]. Three, two-item subscales are positively worded: Self-Kindness, Common Humanity, and Mindfulness, and three are negatively worded: Isolation, Over-Identification, and Self-Judgment. Items are rated on a 5-point Likert scale ranging from 1 = almost never to 5 = almost always. A total score is calculated by adding all 12 items with reverse scoring applied to negatively worded items. Higher scores reflect greater self-compassion. Total scores range from 12 to 60 with a theoretical median of 30. Raes et al. [50] reported an overall Cronbach’s alpha of 0.87 for the 12-item SCS-SF and a correlation of 0.97 with the long form. 

Difficulties in Emotion Regulation Scale (DERS; [48]). The DERS is a self-reported measure that assesses emotional regulation difficulties. Non-acceptance of emotional responses (six items), lack of emotional awareness (six items), lack of emotional clarity (five items), difficulties engaging in goal-directed behaviour (five items) and limited access to emotion regulational strategies (eight items) were included. All items were answered on a five-point response scale from 1 = Almost never to 5 = Almost always, with higher scores suggesting more difficulties in the use of each emotional regulation strategy. Scores could range from 6 to 30 for Non-acceptance of emotional responses and lack of emotional awareness with a theoretical median of 18. The possible range for lack of emotional clarity and difficulties engaging in goal-directed behaviour was 5 to 25 (theoretical median 15). Limited access to emotional regulation strategies scores had a possible range of 8 to 40 (theoretical median 24). Non-acceptance was reverse-coded as adaptive items of acceptance. All other subscales were included as maladaptive emotional regulation strategies.

Emotion Regulation Questionnaire (ERQ; [47]). The ERQ is a 10-item measure assessing the emotional regulation strategies of Expressive Suppression (ES; four items, e.g., ‘I keep my emotions to myself’) and Cognitive Reappraisal (CR; six items), e.g., ‘When I want to feel less negative emotion, I change the way I am thinking about the situation’). Respondents rate on a seven-point Likert scale from 1 = strongly disagree to 7 = strongly agree. Scores on CR can range from 6 to 42 (theoretical median 21) and ES scores can range from 4 to 28 (theoretical median 14). Gross and John [47] reported good Cronbach’s alpha reliabilities for both scales across four samples (ES averaged 0.73 and CR averaged 0.79) and the test–retest reliability was 0.69 over three months, as well as good construct validity.

Brief COPE scale—Problem-focused coping subscale (Brief-COPE; [46]). The Brief-COPE problem-focused subscale is an eight-item self-report subscale that measures coping strategies aimed at changing the stressful situation. All items are answered on a 4-point response scale from 1 = ‘I haven’t been doing this at all’ to 4 = ‘I have been doing this a lot’, indicating how much they perceive themselves to be using each strategy including active coping, planning, and using information supports. For this study, two items measuring positive reframing were removed to avoid overlap with the ERQ assessment of cognitive reappraisal. Therefore, six items from the problem-focused subscale were used. The possible range was from 6 to 24 and the theoretical median was 12.

Cognitive Emotion Regulation Questionnaire—Rumination subscale (CER [49). The CERQ Rumination scale holds four items that assess cognitive rumination (e.g., “I am preoccupied with what I think and feel about what I have experienced”). Items are measured on a five-point response scale 1 = Almost never to 5 = Almost always, with higher scores indicating greater frequency of use of rumination. Total scores can range from 4 to 20 with a theoretical median of 12. The empirically derived measure of rumination developed by Chesney et al. [24] has sound psychometric properties including good factorial validity and high reliability with alphas ranging between 0.75 and 0.87.

### 2.3. Data Analysis

Prior to the analyses, data from all measures were checked for missing data, univariate and multivariate outliers, skewness, and kurtosis (see Table 2). All variables appeared normally distributed with no skewness or kurtosis and no outliers. While there was some missing data, this did not exceed five percent of responses on any measure and, therefore, mean substitution was used for missing data [51]. Following the precedent from previous research [52], emotional awareness and emotion clarity were combined into a composite variable of emotional understanding. Emotional understanding comprised 11 items with higher scores indicating greater difficulties in understanding emotions. Scores could range from 11 to 55 (theoretical mean 33). This provides a more reliable measure of emotional understanding and reduces the number of mediators being tested in the models. After these analyses, relationships among the measures were examined using bivariate Pearson product-moment correlations. The mediation analyses were conducted using the PROCESS V4.2 (model 4) macro for SPSS Version 29 [53]. The models used five thousand bootstrapped samples and percentiles-base ninety-five percent confidence intervals. Three parallel mediation models were used to assess the indirect effects of maladaptive and adaptive emotion regulation on self-compassion relationship with the three mental health outcomes in perinatal women (anxiety, depression, and social anxiety). Further prevalence analyses were conducted to assess the percentage of perinatal women in the overall sample with scores above the clinical cut-off scores for HADS anxiety and depression (>11; [40]) and SIPS (>12; [43]) scores as well as the percentage of women engaged in therapy. Respectively, 93.2%, 70.9% and 65.7% of women presented with clinically significant anxiety, depression, and social anxiety scores. Similarly, the HADS and SIPS mean scores sat well above the clinical cut-off, highlighting a largely clinical sample.

A multivariate analysis of variance (MANOVA) was used to compare current, recent or no engagement in therapy on mental health outcomes. MANOVA indicated differences across the three levels of therapy engagement groups for anxiety, depression, and social anxiety (Pillai’s trace = 0.058, *F*(6, 518) = 2.57, *p* < 0.05, partial eta squared = 0.030. Univariate tests showed that the group difference was confined to scores on anxiety (*F*(2, 260) = 6.21, *p* < 0.01, partial eta squared = 0.046). Student Neuman Keul’s post hoc analysis confirmed that currently being in therapy was associated with a significant increase in anxiety scores in comparison to not being in therapy. Therefore, therapy was included as a covariate in the mediation analyses.

## 3. Results

### 3.1. Correlation Analyses

As hypothesised (H1), the bivariate correlations (see Table 3) indicated that anxiety, depression and social anxiety measures (HADS and SIPS) had strong negative associations with self-compassion and the adaptive emotion regulation measures (acceptance, cognitive reappraisal, problem solving). In addition, maladaptive emotion regulation measures (lack of emotional understanding, expressive suppression, rumination, lack of goal-directed behaviour and lack of emotional regulation strategies) had strong positive associations with anxiety, depression, and social anxiety. High COVID-19 perceived experience in postnatal women had a weak positive correlation with anxiety and depression but not social anxiety. Additionally, higher COVID-19 perceived experience in postnatal women (*n* = 235) had a strong negative correlation with self-compassion. COVID-19 perceived experience in pregnant women failed to significantly correlate with any mental health outcomes.

### 3.2. Multiple Mediation Model

**Figure 1:**
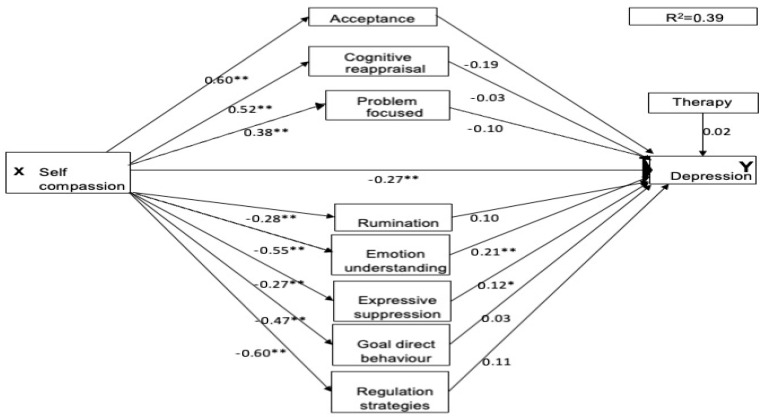
Parallel Mediation model for depression. Note: * = *p* < 0.05, ** *p* < 0.01.

**Figure 2:**
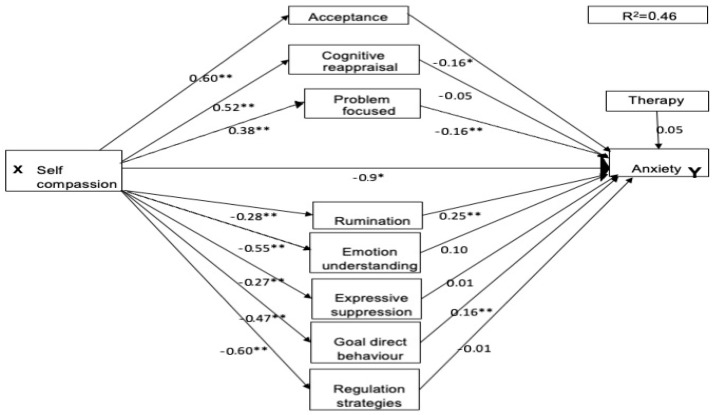
Parallel mediation model for anxiety. Note: * = *p* < 0.05, ** *p* < 0.01.

**Figure 3:**
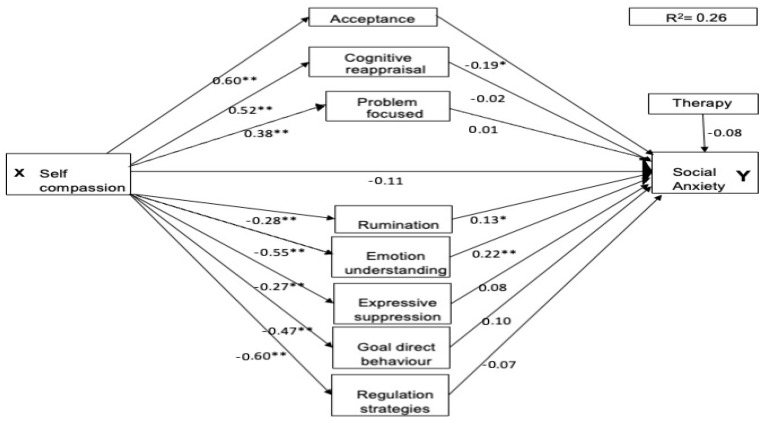
Parallel mediation model for social anxiety. Note: * = *p* < 0.05, ** *p* < 0.01.

As two participants did not specify their therapy experience, the three parallel mediation analyses each included 263 participants. For all three mental health conditions, the pathways between self-compassion and each of the emotional regulation strategies were significant. Higher self-compassion directly predicted lower maladaptive emotion regulation scores and higher adaptive emotion regulation scores (see Figure 1, Figure 2 and Figure 3 for standardised coefficients). For the adaptive pathways, self-compassion was a significant positive predictor for acceptance (a1, β = 0.60, *p* < 0.001, 95% CI [3.81, 5.38]), cognitive reappraisal (a2, β = 0.52, *p* < 0.001, 95% CI [3.70, 5.67]) and engagement in problem-focused strategies (a3 = 0.38, *p* < 0.001, 95% CI [1.74, 3.32]). For the maladaptive pathways, it was also a significant negative predictor for all pathways rumination (a4, β = −0.28, *p* < 0.001, 95% CI [−0.43, −0.18]), lack of emotional understanding (a5, β = −0.55, *p* < 0.001, 95% CI [−6.49, −4.35]), expressive suppression (a6, β = −0.27, *p* < 0.001, 95% CI [−2.65, −0.99]), lack of goal-directed behaviour (a7, β = −0.47, *p* < 0.001, 95% CI [−3.19, −1.96]), and lack of access to regulation strategies (a8, β = −0.61, *p* < 0.001, 95% CI [−6.13, −4.36]). The therapy covariate was non-significant for all mental health conditions.

The total effect of self-compassion was significant for depression (c, β = −0.55, *p* < 0.001, 95% CI [−3.38, −2.27]), anxiety (c, β = −0.57, *p* < 0.001, 95% CI [−4.03, −2.77]), and social anxiety (c, β = −0.42, *p* < 0.001, 95% CI [−9.85, −5.54]).

### 3.3. Prediction of Depression

As shown in Table 4, as a direct predictor, self-compassion strongly predicted lower scores of depression symptoms (c’, β = −0.27, *p* < 0.01, 95% CI [−2.20, −0.52]) in the perinatal sample and was partially mediated by the total indirect effect of the mediator variables (β = −0.29, *p* < 0.01, 95% CI [−0.42, −0.16]). The specific maladaptive strategy of Lack of emotional understanding also predicted depression (b1, β = −0.21, *p* < 0.001, 95% CI [0.05, 0.18]) and the mediation effect was significant (β = −0.12, *p* < 0.001, 95% CI [−0.19, −0.05]). The maladaptive strategy of expressive suppression was also a modest predictor of depression (b1, β = 0.12, *p* < 0.05, 95% CI [0.01, 0.18]). However, the mediation effect was marginally non-significant (β = −0.04, *p* < 0.05, 95% CI [−0.08, 0.00]). Interestingly, rumination, goal-directed behaviour, access to regulation strategies and all adaptive emotional regulation strategies were non-significant mediators for depression (refer to Figure 1 and Table 4).

### 3.4. Prediction of Anxiety

Although a modest effect, self-compassion directly (c’, β = −0.19, *p* < 0.05, 95% CI [−2.07, −0.24]) predicted reduced anxiety symptoms. Four of the mediators also predicted anxiety: scores: rumination (b1, β = 0.25, *p* < 0.001, 95% CI [0.83, 2.03]), goal-directed behaviour (b1, β = 0.16, *p* < 0.01, 95% CI [0.04, 0.31]), problem-focused strategies (b1, β = −0.16, *p* < 0.01, 95% CI [−0.24,−0.04]) and acceptance (b1, β = −0.16, *p* < 0.05, 95% CI [−2.20, −0.52]). As shown in Table 4, the total indirect mediation effect was significant (β = −0.37, *p* < 0.01, 95% CI [−0.51, −0.25]) and mediation was significant for these four mediators. Thus, when problem-focused strategies and acceptance were higher, the reduction in anxiety symptoms by self-compassion was enhanced. Alternatively, when high rumination and a lack of goal-directed strategies were reported, anxiety scores increased and the ameliorative influence of self-compassion was reduced. Access to emotional regulation strategies, expressive suppression, emotional understanding, and cognitive reappraisal were non-significant mediators for anxiety scores (Table 4). Figure 2 presents the parallel mediation model for anxiety.

### 3.5. Prediction of Social Anxiety

Unlike depression and anxiety, self-compassion was fully mediated as a predictor of social anxiety scores (see Table 4 and Figure 3; c’, β = −0.11, *p* = 0.21, 95% CI [−5.38, 1.20]). The total indirect effect was significant (see Table 3, β = −0.31, *p* < 0.01, 95% CI [−0.45, −0.16]); three of the mediators predicted anxiety scores: Lack of emotional understanding (b1, β = −0.22, *p* < 0.01, 95% CI [0.16, 0.68]); rumination (b1, β = 0.13, *p* < 0.05, 95% CI [−0.21, −0.05]) and Acceptance (β = −0.11, *p* < 0.05, 95% CI [−0.22, −0.01]). Of these three mediators, lack of emotional understanding (β = −0.12, *p* < 0.05, 95% CI [0.20, 4.44, and acceptance were significant]) (b1, β = 0.13, *p* < 0.05, 95% CI [0.20, 4.44]). Rumination was a borderline significant mediator (β = −0.04, *p* < 0.05, 95% CI [−0.08, 0.00]). The other potential mediators of cognitive reappraisal, problem-focused strategies, expressive suppression, lack of goal-directed behaviour and regulation strategies all failed to predict social anxiety scores or contribute to mediation (see Table 4).

## 4. Discussion

There are many determinants that increase the risk of mental health concerns for perinatal women including but not limited to environmental stressors, bodily and hormonal changes, sleep deprivation and fear of childbirth [1,2,9,10,54,55]. The present study examined the less explored factors including self-compassion, emotional regulation strategies, and the COVID-19-perceived experience as predictors of perinatal anxiety, depression and social anxiety. As expected, (hypothesis one), and consistent with non-perinatal research [22,24,26], increased self-compassion and adaptive emotional regulation factors correlated with lower symptoms of depression, anxiety and social anxiety. Alternately, and in partial support of the second hypothesis, lower self-compassion and greater maladaptive emotion regulation related to higher depression, anxiety, and social anxiety symptoms in perinatal women. The mediation hypotheses were also partially supported with unique adaptive and maladaptive emotional regulation strategies mediating the influence of self-compassion for depression, anxiety and social anxiety. For depression symptoms, a lack of emotional understanding, and expressive suppression were positive mediators. For anxiety symptoms, rumination, goal-directed behaviour, acceptance and problem-focused strategies were significant mediators. For social anxiety symptoms, acceptance, lack of emotional understanding, and rumination were significant mediators. Interestingly, whereas self-compassion’s effect was partially mediated for anxiety and depression, it was fully mediated for social anxiety.

Despite being a broad community sample of perinatal women, there was a high representation of clinical levels of self-reported depression (70.9%), anxiety (93.2%) and social anxiety (65.7%). Notably, women who were engaged in therapy had significantly higher general anxiety scores than those who had not received psychological treatment in the last two years. Taken together, these findings emphasise the need to better understand the specific factors that either exacerbate or alleviate perinatal mental health issues to better support perinatal women in therapy. This discussion focuses on the finding that the COVID-19 experience did not influence depression, anxiety, and social anxiety, and the unique emotional regulation strategies that mediated the effects of self-compassion on perinatal mental health outcomes. The final section discusses methodological considerations and directions for further research.

### 4.1. The Influence of the COVID-19 Pandemic Experience

Despite widespread concerns regarding the potential impact of the COVID-19 pandemic on perinatal mental health [5,6,7,8], in our study, the perceived experience of COVID-19 did not correlate significantly with social anxiety in either pregnant or postnatal women. Furthermore, there was only a weak association with depression and anxiety, and these associations were observed solely in postnatal women. This suggests that the heightened mental health concerns observed in perinatal women during their postnatal phase persisted during the pandemic, but this was not necessarily attributable to their COVID-19 experiences in pregnancy and post-birth. As the data were collected in the later stages of the pandemic, it may also be that the sense of threat from COVID-19 had dissipated at the time the women completed the survey and so had a less intense influence on the relationship between self-compassion and mental health outcomes. In Australia, the height of concern in the pandemic was from 2020 to 2021 when state borders were closed, and there were numerous lockdowns as the country effectively limited cross infections and deaths. The hospital system was also overcrowded at threat time and without vaccines, many feared attending [12]. However, with the introduction of vaccines and reduced restrictions in 2022, while deaths from COVID-19 increased, community concern and problems of access to facilities eased [12]. As our data collection began in 2022, recollection effects may have influenced the scores of the COVID-19 measures. Excluding the COVID-19 experience measure from the parallel mediation analyses permitted a focus on how specific emotional regulation strategies mediated the predictive relationship between self-compassion and perinatal mental health outcomes.

### 4.2. Specific Predictors of Perinatal Depression

The results for perinatal depression aligned somewhat with expectations, revealing that self-compassion predicted depression symptoms both directly and indirectly. This mirrors the findings of Carona et al. [28] that higher self-compassion directly predicted lower depression symptoms in perinatal women. To build on these findings, future research could look at specific self-compassion features to identify whether self-kindness (vs. self-judgement), awareness of common humanity during challenges (vs. feeling isolated in suffering), or engagement in mindfulness (vs. overidentification with difficult experience; [29]) are more important for reducing postnatal depression.

The current study also identified emotional regulation strategies that were the most influential mechanisms of the influence of self-compassion on perinatal depression. This extends Corona et al.’s finding that general deficits in emotion regulation mediated the effect of self-compassion on perinatal depression. The specific emotional regulation strategy identified as a significant mediator was lack of emotional understanding with expressive suppression as a predictor of depression and close to significant as a mediator. The lack of emotional understanding subscale comprises both a lack of awareness of emotions as they are occurring and clarity about which emotion is being experienced. In addition, Expressive suppression is a form of experiential avoidance that exacerbates distress through attempts to inhibit the expression of emotion [20]. Therefore, the more that perinatal women are aware and clear on the emotion they are experiencing and the more they can express that emotion, the greater it would seem will be the benefits of self-compassion in reducing depression. This is because self-compassion helps the person to recognise suffering and take action to alleviate it [29].

Contrary to other research (e.g., [56]), rumination did not predict depression in this sample, despite being known as a prominent maintaining feature in depression [57]. It is possible that the limited number of items in our measure may not have covered all relevant features of rumination in depression and so was less representative of depressive rumination. Moreover, the cognitive emotion regulation questionnaire (CERQ; [49]) rumination subscale items such as “I dwell on the feelings the situation has evoked in me” could have been interpreted as worrying about emotions, which is more in line with anxiety than depression. Supporting this possibility, rumination did significantly predict anxiety in this study. To better understand the effect rumination has on the relationship between self-compassion and perinatal depression, future research could use a more comprehensive measure of rumination to fully test the role of rumination in perinatal women (e.g., Ruminative Responses Scale (RRS; [58,59]). As they stand, our findings suggest that it is most useful to focus on improving emotional understanding and reducing expressive suppression to enhance the effect of self-compassion on perinatal depression.

### 4.3. Specific Predictors of Perinatal Anxiety

Consistent with our hypotheses, and findings from preliminary perinatal research [26,36], self-compassion directly predicted perinatal anxiety symptoms. This finding supports Felder et al. [26] who found that 10–24% of variance in perinatal anxiety could be explained by self-compassion. The direct pathway between self-compassion and anxiety, however, exhibited robust mediators that were different from those identified for depression. goal-directed behaviour, and problem-focused strategies were mechanisms of the influence of self-compassion on anxiety. Unlike self-compassion’s relationship with depression, rumination also predicted depression and was borderline non-significant as a mediator of its influence such that rumination reduced the effect self-compassion had on lowering anxiety. As previously mentioned, our rumination measure’s items may align with features of worry as well as rumination, and this may account for the heightened influence of rumination on anxiety within this sample. Considering that worry constitutes a fundamental aspect of anxiety ([60]), it follows logically that elevated levels of worry could impede perinatal women’s capacity to cultivate self-compassion as a means of mitigating anxiety symptoms. Importantly, future research could better understand how rumination predicts perinatal anxiety by using a measure of worry (e.g., the Penn State Worry Questionnaire, [61]) and a comprehensive rumination scale. Moreover, our data suggest that directing awareness towards goal-oriented and problem-focused strategies might enhance the effects of self-compassion and alleviate anxiety symptoms by providing women with a heightened sense of control over prevailing issues during this critical period. Acceptance of anxiety symptoms will also help alleviate concerns about emotional states, creating a mental space conducive to the cultivation of self-compassion.

Referring to Neff’s [29] model of self-compassion, it follows that if perinatal women increase their focus on ways of solving problems and directing thoughts toward goals that support them and their baby this will enhance experiences of self-kindness, mindfulness and normalising their perinatal experience as part of common humanity. Likewise, building acceptance of their unique experiences as soon-to-be or new mothers may reduce worries regarding the unknown and fears of motherhood, whereas ruminating over them would only increase the presence of anxiety about being a “good mother” [19]. Taken together, these findings provide unique insights into emotional regulation strategies that might best support perinatal women to prevent and reduce perinatal anxiety during such a valuable time in their lives.

### 4.4. Novel Insights into Perinatal Social Anxiety

To the best of our knowledge, our study is the first exploration of social anxiety in perinatal women. Notably, 65.7% of our sample surpassed the clinical cut-off for social anxiety scores on the SIPS. This alone underscores the necessity for a deeper investigation of the influence of social anxiety among perinatal women, considering the substantial social transformations experienced by women during this period in their lives [19].

Unlike the predictive pathways for anxiety and depression, self-compassion’s effect on social anxiety was fully mediated by emotional regulation strategies. This was unexpected as self-compassion was found to be partially mediated in predicting social anxiety within a general community sample [22]. Respectively, a lack of emotional understanding and increased rumination predicted greater social anxiety symptoms and higher acceptance had a negative effect on social anxiety with acceptance and understanding as significant mediation pathways for self-compassion and rumination close to being a third significant pathway. The items of the non-acceptance of emotions scale capture negative evaluations of the self for experiencing distress (e.g., “When I’m upset, I feel ashamed of myself for feeling that way”). Such experiences of shame and self-criticism for having social anxiety are major features of the condition [25]. Thus, similarly to depression, the effects of compassion toward the self in reducing social anxiety will be enhanced when the person has a greater level of acceptance of the emotion they are experiencing and a clear understanding of the emotion. In addition, reducing rumination enhances the benefits of self-compassionate responses to distress. These findings highlight the gap in our understanding of how social anxiety presents for perinatal women and the benefits of constructive emotional regulation strategies and self-compassionate responses for perinatal women experiencing social anxiety.

### 4.5. Methodological Considerations and Directions for Future Research

A strength of this study was the relatively large, diverse, and widespread perinatal sample drawn from across Australia. However, using a convenience and heterogeneous sampling method limits the capacity to generalise the results to the overall population of perinatal women. Also, our data were drawn from a largely clinical sample of perinatal women, and this may have raised the levels of depression, anxiety and social anxiety symptoms relative to the general population. In addition, the use of cross-sectional data means our findings cannot determine causality in the identified relationships. Nevertheless, given this was an exploratory study, the data will assist in framing future longitudinal research with more generally representative samples of perinatal women.

Given that specific emotional regulation strategies in the perinatal population had not been explored before, this study chose to focus on well-researched emotional regulation strategies with an introspective focus. Future research could include analyses for exteroceptive emotional regulation strategies such as behavioural avoidance. This would extend our knowledge of possibly helpful (or unhelpful) behavioural regulation strategies to support self-compassion and mental health outcomes. Moreover, the use of self-report measures poses a risk of results being influenced by participant honesty, bias, and introspective ability. To reduce this risk, in this study, only well-researched and validated measures used with the perinatal population and for assessing mental health outcomes were included. Nevertheless, self-reported measures assume that the measurement of variables is reliable and valid. Thus, measurement errors can lead to biased estimates and affect the accuracy of the model. Future research could look to incorporate structural equation modelling techniques which can control for measurement error by including error terms in analyses and/or combining measures into latent variables both of which reduce the effects of measurement error [62]. Future work could also look to confirm the findings through observational and single-case study research with the use of state and trait measures.

## 5. Conclusions

In conclusion, this study provides novel and unique insights into the influence of specific emotional regulation strategies, including self-compassion, and directional and predictive relationships for perinatal mental health outcomes. Importantly, we found that different emotional regulation strategies were important for different mental health issues. This highlights the need for further work on how emotional regulation strategies can be incorporated into interventions for the prevention and treatment of perinatal depression, anxiety, and social anxiety.

## Figures and Tables

**Table 1:** Demographic characteristics of the sample.

Demographic Variable	M (SD) or % (*n*) or Range
Age	31.17 (5.27) Minimum = 18 years; Maximum = 40 years
Perinatal status	
Pregnant	11.3% (30)
Postnatal	83.5% (221)
Not specified	5.3% (14)
Number of children	1.88 (0.33)
Therapy status	
Currently	38.1% (101)
Recently (last two years)	23.8% (63)
No therapy	37.4% (99)
Not specified	0.8% (2)
Relationship status	
Married/De facto	89.1% (236)
Single	9.8% (26)
Divorced	0.4% (1)
Not specified	0.8% (2)
Career status	
Employed full-time	21.1% (56)
Employed part-time	27.2% (72)
Employed casually	6.8% (18)
Studying full time	12.8% (34)
Studying part-time	27.2% (72)
Maternity leave	28.3% (75)
Unemployed	6.0% (16)

**Table 2:** Descriptive statistics, skewness, kurtosis, and Cronbach’s alpha reliability coefficients.

	α	M	SD	Skewness	Kurtosis
COVID-19-PPQ—Pregnancy (*n* = 30)	0.79	14.23	5.20	−0.75	1.50
COVID-19-PPQ—postnatal (*n* = 235)	0.67	16.07	4.44	0.27	0.13
HADS Anxiety	0.85	17.87	4.37	0.18	−0.38
HADS Depression	0.80	13.75	4.718	0.67	0.59
SIPS	0.95	19.96	13.32	0.58	−0.44
Self-Compassion Scale	0.87	2.67	0.72	0.26	−0.05
DERS	0.92				
Acceptance of emotions	0.89	18.88	5.54	0.03	−0.43
Goal-directed behaviour	0.80	16.64	3.95	−0.09	−0.50
Emotional regulation strategies	0.87	23.61	6.26	−0.03	−0.17
Emotion clarity	0.73	13.23	3.41	0.26	−0.28
Emotion awareness	0.85	14.49	4.62	0.14	−0.55
Emotional Understanding	0.69	27.72	7.13	0.04	−0.46
ERQ	0.76				
Expressive suppression	0.75	14.38	4.83	0.41	−0.21
Cognitive reappraisal	0.87	25.56	6.51	0.22	−0.39
Brief COPE-Problem-focused strategies	0.87	23.75	4.86	−0.33	−0.29
CERQ—Rumination	0.78	3.58	0.77	−0.61	0.76

Note. N = 265.

**Table 3:** Bivariate Correlations for all variables.

	1	2	3	4	5	6	7	8	9	10	11	12	13	14
1. COVID-19-perceived experience—Pregnancy	-	-	−0.07	0.35	−0.03	−0.34	0.03	−0.14	−0.15	0.20	0.18	0.03	−0.40 *	−0.00
2. COVID-19-perceived experience—Postnatal	-	-	0.17 **	0.16 *	0.04	−0.25 **	−0.08	0.11	0.15 *	0.12	−0.06	−0.20 **	−0.03	0.12
3. HADS Anxiety			-	0.54 **	0.48 **	−0.58 **	−0.49 **	0.46 **	0.50 **	0.38 **	0.19 **	−0.34 **	−0.23 **	0.41 **
4. HADS Depression				-	0.35 **	−0.58 **	−0.37 **	0.33 **	0.45 **	0.49 **	0.37 **	−0.33 **	−0.28 **	0.23 **
5. SIPS					-	−0.40 **	−0.38 **	0.31 **	0.35 **	0.39 **	0.24 **	−0.21 **	−0.13 *	0.23 **
6. Self Compassion Scale						-	0.60 **	−0.48 **	−0.61 **	−0.52 **	−0.23 **	0.53 **	0.28 **	−0.34 **
DERS														
7. Acceptance							-	−0.50 **	−0.72 **	−0.36 **	−0.30 **	0.17 **	0.09	−0.29 **
8. Goal-directed behaviour								-	0.62 **	0.28 **	0.15 *	−0.24 **	−0.06	0.35 **
9. Access to emotional regulation strategies									-	0.42 **	0.31 **	−0.30 **	−0.15 *	0.38 **
10. Emotional Understanding										-	0.36 **	−0.39 **	−0.35 **	0.03
ERQ														
11. Expressive suppression											-	0.07	−0.14 *	0.07
12. Cognitive reappraisal												-	0.30 **	−0.14 *
Brief COPE														
13. Problem-focused coping													-	0.16 **
CERQ														-
14. Rumination														

Note. ** *p* < 0.01, * *p* < 0.05.

**Table 4:** Sandardised coefficients and confidence levels for total direct, total indirect and individual mediation pathways.

	Depression	Anxiety	Social Anxiety
Total direct	0.27 ** [2.20, 0.52]	0.19 * [2.07, 0.24]	0.11 [5.38, 1.20]
Total Indirect	0.29 * [−0.42, 0.16]	0.37 * [−0.51, −0.25]	0.31 ** [−0.45, −0.16]
Acceptance of emotions	0.03 [−0.08, 0.13]	0.09 * [−0.19, −0.01]	−0.11 * [−0.22, −0.01]
Cognitive reappraisal	−0.02 [−0.08, 0.05]	0.02 [−0.09, 0.04]	−0.01 [−0.09, 0.07]
Problem-focused strategies	−0.04 [−0.10, 0.01]	−0.06 * [−0.11, −0.01]	0.00 [−0.05, 0.06]
Rumination	−0.03 [−0.07, 0.01]	−0.07 * [−0.12, −0.02]	−0.04 [−0.08, 0.00]
Emotional understanding	−0.12 * [−0.19, −0.05]	−0.05 [−0.12, 0.01]	−0.12 * [−0.21, −0.05]
Expressive suppression	−0.04 [−0.08, 0.00]	0.00 [−0.03, 0.03]	−0.02 [−0.06, 0.02]
Goal-directed behaviour	−0.01 [−0.08, 0.05]	−0.07 * [−0.14, −0.01]	−0.05 [−0.11, 0.02]
Emotional regulation strategies	−0.07 [−0.17, 0.03]	0.01 [−0.09, 0.04]	−0.01 [−0.08, 0.16]

Note; N = 265. * = *p* < 0.05, ** *p* < 0.01.

## Data Availability

The data supporting the conclusions of this article can be provided by the corresponding author upon reasonable request and university ethics approval.
